# Evidence for feasibility of fetal trophoblastic cell‐based noninvasive prenatal testing†

**DOI:** 10.1002/pd.4924

**Published:** 2016-10-02

**Authors:** Amy M. Breman, Jennifer C. Chow, Lance U'Ren, Elizabeth A. Normand, Sadeem Qdaisat, Li Zhao, David M. Henke, Rui Chen, Chad A. Shaw, Laird Jackson, Yaping Yang, Liesbeth Vossaert, Rachel H. V. Needham, Elizabeth J. Chang, Daniel Campton, Jeffrey L. Werbin, Ron C. Seubert, Ignatia B. Van den Veyver, Jackie L. Stilwell, Eric P. Kaldjian, Arthur L. Beaudet

**Affiliations:** ^1^ Department of Molecular and Human GeneticsBaylor College of MedicineHoustonTXUSA; ^2^ RareCyte, Inc.SeattleWAUSA; ^3^ Department of Obstetrics and GynecologyBaylor College of MedicineHoustonTXUSA; ^4^ Department of Obstetrics and GynecologyDrexel University College of MedicinePhiladelphiaPAUSA; ^5^ Texas Children's HospitalHoustonTXUSA

## Abstract

**Objective:**

The goal was to develop methods for detection of chromosomal and subchromosomal abnormalities in fetal cells in the mother's circulation at 10–16 weeks' gestation using analysis by array comparative genomic hybridization (CGH) and/or next‐generation sequencing (NGS).

**Method:**

Nucleated cells from 30 mL of blood collected at 10–16 weeks' gestation were separated from red cells by density fractionation and then immunostained to identify cytokeratin positive and CD45 negative trophoblasts. Individual cells were picked and subjected to whole genome amplification, genotyping, and analysis by array CGH and NGS.

**Results:**

Fetal cells were recovered from most samples as documented by Y chromosome PCR, short tandem repeat analysis, array CGH, and NGS including over 30 normal male cells, one 47,XXY cell from an affected fetus, one trisomy 18 cell from an affected fetus, nine cells from a trisomy 21 case, three normal cells and one trisomy 13 cell from a case with confined placental mosaicism, and two chromosome 15 deletion cells from a case known by CVS to have a 2.7 Mb de novo deletion.

**Conclusion:**

We believe that this is the first report of using array CGH and NGS whole genome sequencing to detect chromosomal abnormalities in fetal trophoblastic cells from maternal blood. © 2016 The Authors. *Prenatal Diagnosis* published by John Wiley & Sons, Ltd.

## Abstract 2

What's already known about this topic?

- Analysis of cell‐free DNA for noninvasive prenatal testing (NIPT) is widely practiced, and the frequency of amniocentesis and CVS has decreased.
- However, cell‐free NIPT is not adequate for detecting smaller deletions and duplications with high specificity, sensitivity, and positive predictive value.
- Although fetal nucleated red blood cells and trophoblastic cells are known to be present in the maternal circulation, it has not been possible to develop a reliable cytogenetic cell‐based form of NIPT.

What does this study add?

- Fetal cytotrophoblasts were successfully recovered from maternal blood.
- Although a clinical test has not been validated, for the first time, the feasibility of using array comparative genomic hybridization and next generation sequencing to detect chromosomal and subchromosomal abnormalities is demonstrated.
- The results suggest the possibility of developing a cell‐based form of NIPT with ability to detect abnormalities with a similar accuracy as can currently be obtained with amniocentesis and CVS.

## Introduction

The presence of fetal cells in maternal blood during the first and second trimesters was first described in 1969^1^ and confirmed in 1979,^2^ and the potential to use these cells for prenatal diagnosis was immediately appreciated. Despite extensive efforts focused on recovery of fetal nucleated red blood cells (fnRBCs) followed by fluorescence *in situ* hybridization (FISH) to detect aneuploidy, a collaborative effort reported in 2002 was unable to establish fetal cell‐based analysis as a reliable prenatal clinical test.^3^ In 2001, it was demonstrated that fetal cells could be found in 12 of 12 of women with a normal male pregnancy at 18–22 weeks' gestation,^4^ but first trimester sampling is of greater clinical relevance. Although there is one report in 2012^5^ of successful analysis of trophoblasts in pregnancies at risk of cystic fibrosis or spinal muscular atrophy, this single gene analysis has not been independently replicated. The rapid commercial development and increase in utilization of cell‐free fetal DNA (cffDNA) for noninvasive testing to detect Down syndrome and other aneuploidies have led to a dramatic reduction in the number of amniocentesis and chorionic villus sampling (CVS) diagnostic procedures.^5^, ^6^ With the current limitations of cffDNA assays, this reduction in invasive testing can be predicted to lead to an increased number of births of infants with cytogenetic abnormalities, especially deletions and unbalanced translocations that would have been detected by an invasive test with karyotype or microarray analysis, but are not detected by the current cffDNA analysis.^6^

There are many reports of attempts to recover trophoblasts^7^, ^8^, ^9^, ^10^, ^11^ and fnRBCs^12^, ^13^, ^14^, ^15^ from maternal blood; see Bianchi *et al* for older references.^3^ Attempts to recover male fnRBCs in blood samples obtained prior to CVS or pregnancy termination from women carrying male pregnancies failed in 60–70% of cases^3^, ^16^ leaving some doubt as to whether this cell type is present in sufficient numbers for routine analysis during the first trimester. In contrast, two groups have demonstrated that there are one to six fetal cells per milliliter of mother's blood during the first trimester using very reliable methods for Y chromosome FISH, and showed that these cells are certainly^17^ or most likely^18^ trophoblasts. Based on these reports, we have focused exclusively on detecting trophoblasts. Cytokeratins are long known to be expressed in trophoblasts,^19^ and a cocktail of cytokeratin (CK) antibodies was reported to be effective in staining fetal trophoblastic cells in the maternal circulation.^17^ We recognize that trophoblasts are not technically from the fetus, but just as with CVS, the diagnostic results can be interpreted as being indicative of the fetal genomic status (barring confined placental mosaicism). We use the word fetal in this manuscript to refer to cells having the fetal as opposed to the maternal genome.

Investigators have tried a variety of strategies to enrich circulating fetal cells, including density gradients, immuno‐magnetic bead isolations, fluorescence activated cell sorting (FACS), and filters.^3^, ^5^ Although circulating fetal cells can be recovered, these methods have lacked consistency and repeatability. In addition to the complexities of enrichment, the fetal cells should be retrieved individually, genotyped to exclude maternal cell contamination, and amplified to yield DNA that is of sufficient quality and quantity for genome‐wide analysis. Until now, there is no report of genome‐wide microarray analysis or next‐generation sequencing (NGS) analysis of copy number using fetal cells recovered from maternal blood during the first trimester or early second trimester; both microarray and NGS analyses are reported here. If it were possible to perform high resolution analysis of fetal cells, there is the potential to develop a noninvasive test that could detect smaller deletions and duplications that are not currently reliably detectable using cffDNA analysis.

## Material and Methods

### Sample collection

Blood samples were collected from healthy volunteers from multiple referring centers, following informed consent, according to a protocol approved by Baylor College of Medicine or University of Washington Institutional Review Boards. Participants included women being seen for routine genetic counseling in one of several centers, and in many but not all cases the women were scheduled to undergo a CVS or amniocentesis for prenatal diagnosis via conventional chromosome analysis or array comparative genomic hybridization (CGH) analysis. Blood samples were drawn prior to any procedure. Approximately 30 mL was collected from volunteers into anticoagulant EDTA Vacutainer® tubes (Becton‐Dickinson) with a proprietary preservative (available from RareCyte).

### Fetal gender determination

Maternal plasma was separated from 4 mL of whole blood by two rounds of centrifugation at 3000 ×*g* for 10 min each. The cffDNA was extracted from 500‐μL plasma using the MagNA Pure Compact DNA extraction system, Nucleic Acid Isolation Kit I, Large Volume, according to the manufacturer's instructions (Roche Diagnostics). Gender PCR was carried out on each cffDNA in triplicate on the LightCycler® 480 Real‐Time PCR System (Roche) using Y‐chromosome specific primers and TaqMan probes for the multicopy DYS14 sequence located within the TSPY gene and the single copy SRY gene as described in.^20^ PCR was performed at 95 °C for 3 min, followed by 49 cycles of 95 °C for 15 s, 64 °C for 30 s, with a final 30‐s step at 40 °C. Multiple positive and negative controls were used in each reaction. The fetus was determined to be female if no positive replicates were obtained and male if there were at least two replicates with positive amplifications (Ct of 30–33 for DYS14 and 34–40 for SRY).

### Fetal cell enrichment and staining

Fetal cell enrichment was carried out with RareCyte's trophoblast enrichment and staining method. This has been described for the identification and retrieval of circulating tumor cells (CTCs)^21^ and was modified to allow for retrieval of rare target cells from liquid slide preps. Briefly, 6 mL of preserved blood was added to the AccuCyte® Separation (ACS) tube (RareCyte, Inc., Supporting Information Figure S1A).^21^ In an attempt to reduce the number of slides to be scanned in order to increase the throughput of the test, a step for WBC depletion was explored. In the majority of samples where WBC depletion was performed, RosetteSep Human CD45 and CD36 Depletion Cocktails (STEMCELL Technologies) were combined with the blood for 20 min at room temperature (RT). In some cases a slightly modified WBC depletion protocol was carried out but both gave equivalent WBC depletion and fetal cell recovery results. A float (density 1.058–1.061 g/cm^3^) was then added to the ACS tube. The sample was centrifuged at 5250 ×*g* for 30 min, a sealing ring was applied to the outside of the ACS tube below the WBC band on the float, and the plasma was aspirated. Next 150 μL of a high‐density retrieval fluid (Fluid A) was added, and the ACS tube was centrifuged at 1000 ×*g* for 5 min displacing the less dense WBCs to above the float. A second sealing ring was placed at the top of the float to keep the WBCs in solution above the float. Then 30 μL of 5% paraformaldehyde (Ted Pella, Inc.,) in PBS was added. After 20 min, cells were stained for 1 h at RT by adding a blocking/permeabilizing solution, followed by staining cocktail containing DAPI (BioLegend), anti‐cytokeratin Alexa‐488 (eBiosciences, clone AE1/AE3), anti‐cytokeratin Alexa‐488 (BioLegend clone C11), and anti‐CD45 PE (BioLegend clone 2D1).

### Fetal cell recovery

Following staining, the cells were collected and removed from the antibody cocktail as follows. Briefly, 1 mL of a density increasing reagent (Fluid A) was mixed into the staining solution slurry. The EpiCollector® (RareCyte, Inc.) device was then inserted into the ACS tube and a second fluid of lesser density (Fluid B) was gently layered on top of the denser staining solution slurry. A 1.5‐mL isolation tube containing 200 μL of a 1.1 g/cm^3^ collection fluid (Fluid C), less dense than Fluid B, was inserted into the EpiCollector. The ACS tube, now containing a gradient of density fluids without air gaps, was centrifuged at 1000 ×*g* for 20 min. During centrifugation, cells that have density < 1.1 g/cm^3^ float upward out of the denser fluids into Fluid C within the collection tube. The cells have thus been drawn through the antibody solution, removing unbound antibody in a ‘pseudo‐wash’ process. The isolation tube was then removed from the EpiCollector, 800 μL of PBS was added and stored at 4 °C until imaging.

### Automated image capture and analysis

Stained cells were pipetted into custom well slides (CyteSlide, RareCyte, Supporting Information Figure S1B) that were placed onto the CyteFinder digital scanning microscope to acquire fluorescent images (Supporting Information Figure S1C, S2). The CyteFinder acquired 4‐channel fluorescent images of low magnification (10 × objective) fields of view for each CyteSlide covering the entire well. The high resolution images (40 × objective) of revisited points were imaged with a ‘stack’ of images through the Z plane with 1‐µm steps.

Images were analyzed for the presence of signal above background for CK and CD45 using image analysis software that employs an adaptive auto‐threshold algorithm (RareCyte). Candidate cells that were determined by the algorithm to be CK positive and CD45 negative were presented to the operator by the software for manual confirmation as potential fetal cells.

### Retrieval of individual fetal cells from CyteSlides

Isolation of single cells from CyteSlides was performed with CytePicker® that is part of the CyteFinder instrument. CytePicker is a hydraulically controlled semi‐automated single cell retrieval device that contains three critical parts: (1) needle with 40‐µm bore ceramic tip, (2) pump capable of 10‐nL droplet resolution, and (3) precision Z‐positioning system. After verification that the candidate cell met trophoblastic cell criteria described above, cells were retrieved using a semi‐automated picking routine. During the routine, the glass surface of the CyteSlide is detected with a sensor, the needle is lowered over the cell of interest and a 200‐nL volume is then aspirated to withdraw the cell into the needle tip. To ensure the isolation of single fetal cells without associated WBCs, the initial aspirated volume was dispensed into a cell sorting well in the CyteSlide for repicking to further separate the cell of interest from any co‐aspirated white blood cells. The cell of interest was then re‐picked and dispensed into the bottom of a PCR tube and immediately frozen at −80 °C.

### Whole Genome Amplification and Y‐chromosome specific PCR

Whole genome amplification (WGA) was performed using either the PicoPLEX WGA kit (Rubicon Genomics) or *Ampli*1™ WGA Kit (Silicon BioSystems) in an Applied Biosystems 2720 (ThermoFisher Scientific), a C1000 Touch (Bio‐Rad) or a MyCycler™ Thermal Cycler (Bio‐Rad), according to manufacturers' instructions. To minimize contamination, all WGA reactions were carried out inside a PCR workstation (AirClean Systems) in a pre‐PCR room. PCR products were purified using a DNA Clean Concentrator ^TM^‐25 kit (Zymo Research) according to the manufacturer's instructions. DNA was subsequently eluted into 50 μL of water and stored at −20 °C. The WGA DNA quality and concentration were determined using a Nanodrop spectrophotometer (Nanodrop Inc.).

For male pregnancies, Y‐chromosome specific PCRs were performed to confirm the cells contained the fetal genome. Hemi‐nested PCR was performed in two rounds. Primers Y1.5 and Y1.8 were added at 0.2 μM^22^ with 0.5 mM each dATP, dGTP, dCTP, and 0.25 mM dTTP and dUTP (Promega), Colorless GoTaq Flexi Buffer (Promega), 0.625 units GoTaq polymerase (Promega), and 2 mM MgCl_2_. PCR was performed at 95 °C for 5 min, followed by 40 cycles of 94 °C for 45 s, 56 °C for 30 s, and 72 °C for 30 s, with a final 7‐min extension at 72 °C and hold at 4°C. A nested PCR was performed using 1 μL of the product from the first round of PCR as template but using primers Y1.7 and Y1.8^22^ with the same conditions as described above. Additional PCR tests for the presence of the Y chromosome were performed using the above conditions but using primers to DYS14 or DAZ,^23^ 2.5 mM MgCl_2_, and a 58 °C annealing temperature. PCR products were visualized on EX E‐Gels (ThermoFisher) with E‐Gel 50 bp ladder (ThermoFisher). PCR targeting the X chromosome was also performed (data not shown).

### STR analysis

For cases where the fetus was determined to be female or where Y‐PCR was inconclusive, WGA products from single cells were PCR amplified using a STR kit designed by Silicon Biosystems, which consists of a multiplex primer set targeted to 11 polymorphic tetranucleotide repeats across the human genome. Genomic DNA was also isolated from 1 mL of parental whole blood using the QIAgen DSP Blood Mini Kit (QIAgen) to serve as controls. When available, fetal genomic DNA obtained from either direct or cultured CVS tissue or amniotic fluid was also analyzed. STR was carried out according to the manufacturer's recommendations, and the resulting products were analyzed by capillary electrophoresis. The allele patterns of the WGA products were then compared to the fetal and parental genomic DNA patterns to assess for allelic dropout and expected inheritance patterns. All PCR reactions were prepared in an AirClean PCR workstation. Data analysis was performed using GeneMapper® version 4.0 analysis software (Life Technologies). Any peak with amplitude of less than 1000 RFUs was considered noise and not counted.

### Array CGH

WGA products from single cells were analyzed by array CGH using oligonucleotide‐based SurePrint G3 Human CGH 4x180K arrays from Agilent Technologies as described previously.^24^ Briefly, 1 µg of WGA DNA was labeled per hybridization, using a Nanodrop spectrophotometer for quantification. Because the WGA products ranged in size from 100 bp to 1 kb, it was not necessary to perform DNA fragmentation before labeling. Fetal DNA was labeled with dCTP‐Cy5 and reference DNA was labeled with dCTP‐Cy3, for 3 h at 37 °C using the SureTag DNA Labeling Kit (Agilent). Unincorporated nucleotides were removed using a MultiScreen‐PCRμ96 Filter Plate (Millipore). Hybridizations were carried out at 65 °C for 40–72 h to enhance the binding of WGA DNA. The DNA used as a reference for each fetal cell WGA product was a pool of WGA DNA from multiple (5–10 single cell) WGA reactions from either male or female lymphoblast reference cell lines. Gender‐mismatched references using singe cells amplified with the same WGA method were used.

### Analysis of array data

Slides were scanned into image files using the Agilent G2565 Microarray Scanner. Scanned images were quantified using Agilent Feature Extraction software (v10.10.0.23). Text file outputs containing quantitative data were imported into the Agilent CytoGenomics software (version 2.5.8.11). Data were analyzed using the Aberration Detection Method 2 (ADM2) statistical algorithm at a threshold of 6.0 to identify genomic intervals with copy number changes. To reduce false positive calls, a filter was applied to define the minimum log2 ratio (0.25), the minimum size (100 kb), and the minimum number of probes (100) in a CNV interval. The Derivative Log Ratio Spread (DRLS), a measure of probe to probe noise calculated by the CytoGenomics software, was used as a performance measure for hybridization quality. Array results from circulating fetal cells were evaluated for copy number changes > 1 Mb and were compared to the array results from the corresponding CVS or amniotic fluid samples to determine whether the findings were true positive, true negative, false positive, or false negative. The 100 probe number and 1 Mb interval were chosen arbitrarily at this stage of development to minimize the number of false positive calls because of the noisiness associated with single cell WGA, although the ultimate objective is to detect the smallest possible CNVs with a minimum number of false positives.

### Single cell next‐generation sequencing

In addition to array CGH analysis, we also explored the use of whole shotgun NGS to determine copy number as is commonly applied to embryonic cells for preimplantation genetic screening.^28^, ^29^ We aimed to obtain about 5 million NGS reads per cell. Paired‐end sequencing reads were obtained for each sample. Reads were mapped to human reference genome hg19 using Burrows–Wheeler Aligner (BWA) 25. Base quality recalibration and local realignment were performed using the Genome Analysis Tool Kit (GATK).^26^

To identify copy number variations (CNVs), first the human genome was divided into slide windows according to the control sample. The aligned reads of the control sample were binned into variable‐length windows, and the number of mapped reads in every window was the same. Then the aligned reads of the case sample were counted in each window and the log2 ratio between the case and control was calculated. For trisomy detection, there were 4000 mapped reads within each window, and the total number of windows across the genome was around 2100. The segmentation and copy number estimation was performed using CGHweb R package.^27^

## Results

### Trophoblastic cell enrichment

**Figure 1:**
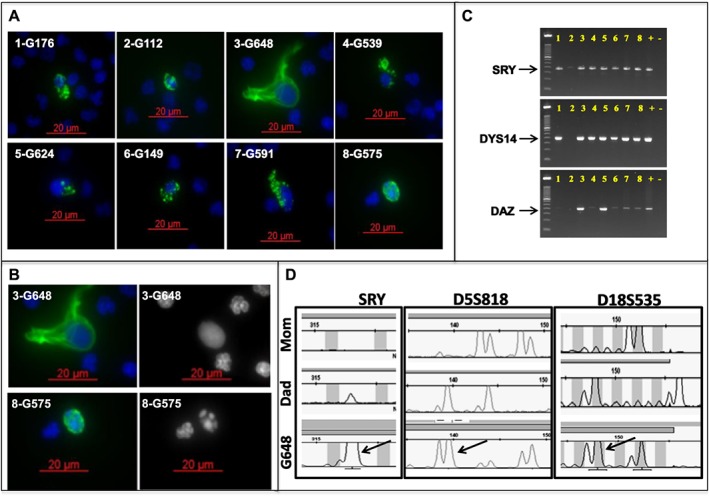
Multiple fetal trophoblastic cells isolated from one patient. A. Eight fetal cells (confirmed by STR analysis) from a single patient (subject 365) showing the range of nuclear (DAPI; blue) and cytokeratin (CK; green) staining morphology. B. Two of the fetal cells from subject 365 demonstrating uniform (top right) and fragmented (bottom right) DAPI staining. C. Hemi‐nested Y‐chromosome specific PCR performed on the eight fetal cells isolated from subject 365 (male fetus), showing positive amplification of one or more targeted regions on the Y chromosome (SRY, DYS14, DAZ). Numbers correspond to the number prefix for each individual cell in A. D. *Ampli*1 STR analysis comparing WGA product from a single fetal cell (G648, subject 365) and genomic DNA from each parent. The three loci shown here demonstrate the expected paternal inheritance (arrows) from the Y chromosome (SRY; left panel) and bi‐parental inheritance from chromosomes 5 (D5S818; center panel) and 18 (D18S535; right panel)

We chose to focus on recovery of trophoblasts that were CK positive based on existing literature,^17^ and we used CD45 staining to exclude most maternal white blood cells. The cells that later proved to carry the fetal genome were completely negative for CD45 staining and had characteristic CK staining that varied from a diffuse cytoplasmic distribution to a micro‐globular appearance (Figure 1A). The nuclear morphology was usually round and smooth with diffuse uniform DAPI staining in contrast to the variegated nuclear staining of mononuclear cells and lobulated nuclei of neutrophils, but occasional fetal nuclei showed fragmentation (Figure 1B). These may represent fetal cells at various stages of degradation within maternal circulation.

### Fetal cell verification

Candidate fetal cells that were subjected to WGA were characterized by Y chromosome specific PCR and genotyping with short tandem repeats (STR) to assess for gender and allelic inheritance patterns (Figure 1C, D). Blood samples from 30 male pregnancies (including one 47,XXY) from multiple collection venues were demonstrated to have cells that were genotypically male by Y‐PCR. For those cases where the fetus was female or Y‐PCR was inconclusive, STR analysis was performed. Allele dropout was common, and only cells with multiple non‐maternal alleles were scored as fetal genome positive (Table 1). The average recovery of circulating fetal cells was 0.74 per milliliter using our initial protocol. After implementing a WBC depletion method to reduce slide numbers and increase sample throughput, the average recovery of fetal cells was 0.36 per milliliter (Table 1) indicating that increased throughput may reduce total fetal cell recovery. For the most part, arrays and NGS were only performed on samples with known abnormalities or on a select few representative male cases to demonstrate feasibility. All others were verified using Y‐PCR or STR only.

**Table 1:** Fetal trophoblastic cell recovery from individual samples

Patient ID	GA	Gender	Depletion	Absolute counts	Counts (cells/mL)	F ‐ M ‐ I/N	Single cell array/NGS result (cells)	Invasive array/Chromosome result
NIPT319	13W0D	Male	No	6 cells/6 mL	1.00	1 ‐ 0 ‐ 5	NL male (1)	NL male
NIPT319	13W0D	Male	Yes	6 cells/6 mL	1.00	3 ‐ 0 ‐ 3	NL male (1)	NL male
NIPT327	10W0D	Male	Yes	9 cells/18 mL	0.50	3 ‐ 0 ‐ 6	ND	ND
NIPT342	13W3D	XXY	Yes	2 cells/9 mL	0.22	1 ‐ 1 ‐ 0	XXY (1)	XXY
NIPT344	15W5D	Female	Yes	5 cells/18 mL	0.28	2 ‐ 0 ‐ 3	ND	ND
NIPT353	12W0D	Male	Yes	8 cells/21.5 mL	0.37	5 ‐ 0 ‐ 3	ND	ND
NIPT359	16W0D	Male	Yes	4 cells/17.5 mL	0.23	4 ‐ 0 ‐ 0	NL male (4)	1.2 Mb del 1q
NIPT365	12W0D	Male	Yes	8 cells/18 mL	0.67	5 ‐ 0 ‐ 3	NL male (4)	ND
NIPT375	13W5D	Female	Yes	3 cells/18 mL	0.17	1 ‐ 1 ‐ 1	Trisomy 18 (1)	Trisomy 18
NIPT386	12W5D	Male	Yes	3 cells/6 mL	0.50	1 ‐ 0 ‐ 2	ND	ND
NIPT392	11W2D	Male	Yes	7 cells/6 mL	1.00	5 ‐ 0 ‐ 2	ND	ND
NIPT402	12W2D	Male	Yes	1 cell/6 mL	0.17	1 ‐ 0 ‐ 0	ND	ND
NIPT414	13W2D	Male	Yes	4 cells/17 mL	0.24	2 ‐ 0 ‐ 2	ND	NL male
NIPT418	12W0D	Male	Yes	3 cells/6 mL	0.50	1 ‐ 0 ‐ 2	ND	ND
NIPT419	10W3D	Male	Yes	1 cell/6 mL	0.33	1 ‐ 0 ‐ 0	ND	ND
NIPT422	12W2D	Male	Yes	3 cells/6 mL	0.50	1 ‐ 0 ‐ 2	ND	ND
NIPT447	13W5D	Male	Yes	5 cells/24 mL	0.21	2 ‐ 2 ‐ 1	Trisomy 21 (2)	Trisomy 21
NIPT476	13W0D	Male	Yes	1 cell/24 mL	0.04	1 ‐ 0 ‐ 0	ND	ND
NIPT479	16W0D	Female	Yes	2 cells/23.5 mL	0.09	2 ‐ 0 ‐ 0	2.7 Mb del 15q (2)	2.7 Mb del 15q
NIPT483	14W1D	Female	Yes	4 cells/24 mL	0.13	2 ‐ 1 ‐ 1	NL female (2)	NL female
NIPT485	13W0D	Male	Yes	1 cell/18 mL	0.06	1 ‐ 0 ‐ 0	ND	ND
NIPT488	11W4D	Female	Yes	1 cell/18 mL	0.06	1 ‐ 0 ‐ 0	Trisomy 21 (1)	Trisomy 21
NIPT490	12W1D	Male	Yes	3 cells/6 mL	0.50	3 ‐ 0 ‐ 0	ND	ND
NIPT493	12W4D	Male	Yes	4 cells/25 mL	0.16	3 ‐ 1 ‐ 0	ND	ND
NIPT495	11W3D	Male	Yes	5 cells/18 mL	0.28	2 ‐ 1 ‐ 2	ND	NL
NIPT497	12W4D	Female	Yes	9 cells/24 mL	0.38	1 ‐ 0 ‐ 8	NL (3)/Trisomy 13 (1)	NL (NIPT Trisomy 13)
NIPT508	16W0D	Male	Yes	3 cells/18 mL	0.17	2 ‐ 0 ‐ 1	NL male (2)	NL male
NIPT511	13W0D	Male	Yes	15 cells/18 mL	0.83	11 ‐ 2 ‐ 2	Trisomy 21 (11)	Trisomy 21
PRI036	12W2D	Male	No	5 cells/4 mL	1.20	5 ‐ 0 ‐ 0	NL Male (2)	ND
PRI041	12W3D	Male	No	2 cells/5 mL	0.50	1 ‐ 0 ‐ 1	ND	ND
PRI042	12W3D	Male	No	3 cells/2.6 mL	1.14	2 ‐ 0 ‐ 1	ND	ND
PRI045	13W2D	Male	No	5 cells/6 mL	0.83	3 ‐ 0 ‐ 2	ND	ND
PRI052	14W0D	Male	Yes	4 cells/5 mL	0.80	2 ‐ 0 ‐ 2	ND	ND
PRI054	12W4D	Male	Yes	2 cells/12 mL	0.17	2 ‐ 0 ‐ 0	ND	ND
PRI37	12W2D	Male	No	2 cells/5 mL	0.40	2 ‐ 0 ‐ 0	NL male (2)	ND
PRI40	11W0D	Male	No	1 cell/7.5 mL	0.13	1 ‐ 0 ‐ 0	NL male (1)	ND

GA, gestational age; F, confirmed fetal genome; M, maternal; I/N, inconclusive/not tested; ND, not done; NL, normal. For NIPT375, STR analysis showed one cell to be fetal, one maternal, and one inconclusive, and only the fetal cell was analyzed.

**Figure 2:**
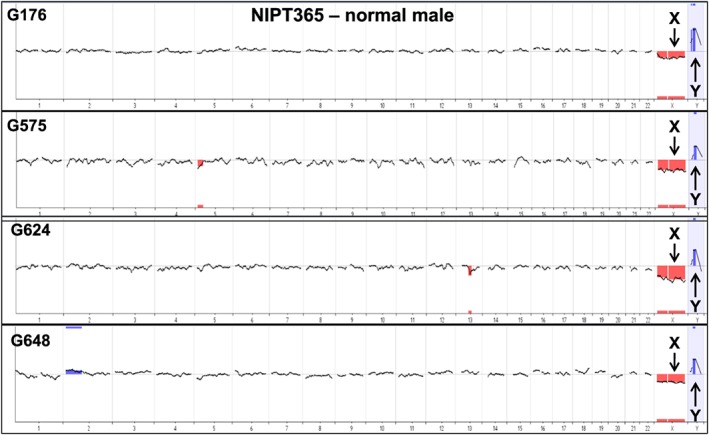
Array CGH on multiple normal male fetal trophoblastic cells from one patient. The arrays all show the expected loss of the X chromosome and gain of the Y chromosome (black arrows) following hybridization with a normal female reference DNA sample. Array data is displayed as a 20 Mb moving average across the genome

**Figure 3:**
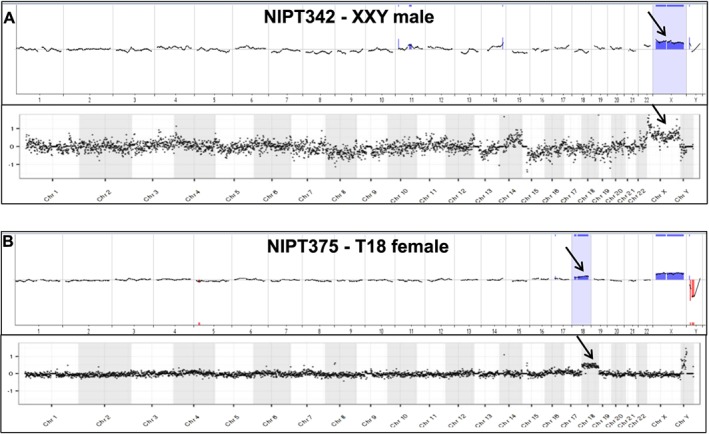
Comparison of array CGH and NGS analyses for two cytogenetically abnormal cases. A. Array CGH whole genome plot (top) and NGS whole genome plot (bottom) on the same WGA product from a single cell derived from a 47,XXY pregnancy (subject 342). Both data plots show the expected gain of the X chromosome (black arrow) when compared to a normal male reference. Both analyses are somewhat noisy, particularly on chromosome 14, although no copy number changes involving chromosome 14 were detected on the clinical array on CVS tissue. B. Whole genome plot (top) and NGS whole genome plot (bottom) from the same WGA product from a single fetal cell derived from a 47,XX,+18 pregnancy (subject 375). Both data plots show the expected gains of chromosomes 18 (black arrows). Additionally, the array plot shows a gain of X and loss of Y because of hybridization with a normal male reference. Array data is displayed as a 20 Mb moving average across the genome. NGS data is displayed as 1000 kb bins across the genome

A subset of cells proven to be fetal genome positive by Y PCR and/or STR genotyping were analyzed using array CGH and/or NGS, including eight cases having a known or suspected cytogenetic abnormality (trisomy 13, trisomy 18, trisomy 21, XXY, and two microdeletions). The sex of the fetus was either known independently from ultrasound or invasive prenatal diagnosis or was determined using Y‐specific PCR of maternal plasma.^20^ We performed array CGH and/or NGS on at least 40 single cells from 16 pregnancies, including 17 normal male cells to show the sex chromosome copy number differences, one 47,XXY, 14 trisomy 21, one trisomy 18, one trisomy 13, 2 cells with a 2.7 Mb deletion on 15q and 4 cells with a 1.2 Mb deletion on 1q (Table 1). Looking first at array CGH data, four cells from a single normal male pregnancy (NIPT365) all demonstrate the expected sex chromosome differences (Figure 2). The gain of copy number for the X chromosome is shown in Figure 3 for a 47,XXY case (NIPT342) sampled at 13 weeks' gestation following a positive cffDNA result for sex chromosome abnormality. The blood sample was obtained prior to a CVS which showed 47,XXY. Also shown in Figure 3 is an array on a single cell from a case (NIPT375) suspected to have a female fetus with trisomy 18 based on cffDNA analysis; a blood sample was obtained from the mother at 13 weeks' gestation prior to a CVS procedure, which confirmed trisomy 18 by karyotype analysis. A microdeletion case was studied following a clinical microarray analysis on a CVS sample that demonstrated a 1.2 Mb deletion of chromosome 1q21.1q21.2 (NIPT359). Array analyses on four individual fetal cells from this case did not detect the abnormality.

### NGS analysis of abnormal samples

**Figure 4:**
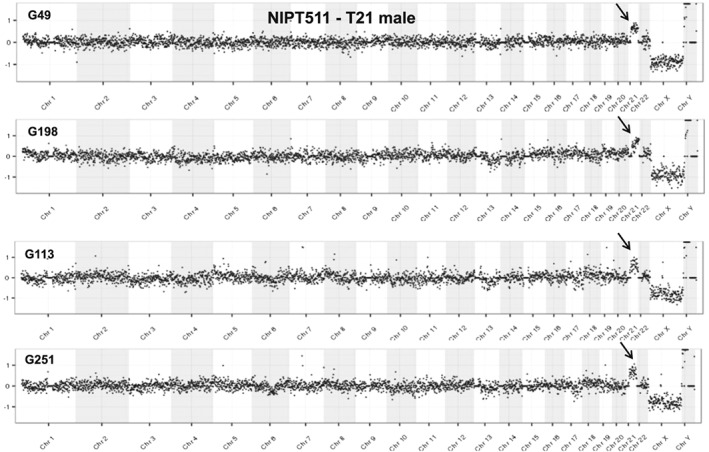
Comparison of NGS analysis of four trisomy 21 cells from a single case. Subject 511 showing four out of eleven cells with similar results. All four plots show the gain of chromosomes 21 (black arrows). Additionally, the plots show a loss of X and gain of Y because of comparison with a normal female reference. NGS data is displayed as 1000 kb bins across the genome

**Figure 5:**
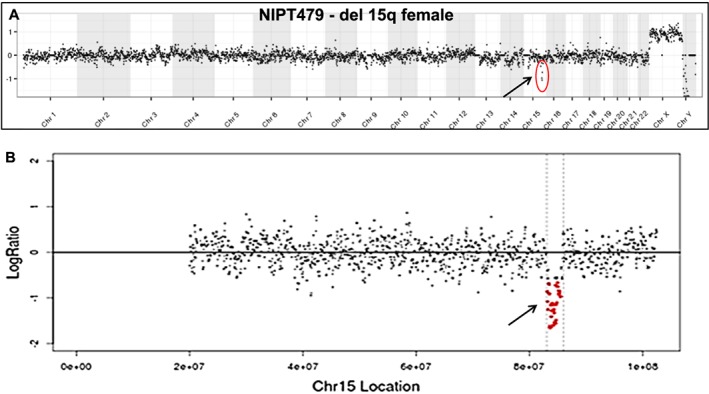
NGS analysis of a fetal trophoblastic cell with a 2.7 Mb deletion of chromosome 15q. A. Whole genome plot from a single cell derived from a fetus harboring a 2.7 Mb deletion of chromosome 15q (subject 479). B. Enlarged plot of chromosome 15 showing the deleted region in red. NGS data is displayed as 1000 kb bins for the whole genome plot and 30 kb bins for the chromosome 15 plot.

Fetal cells were isolated and analyzed by NGS for five pregnancies subsequently proven to have a chromosomal abnormality and one pregnancy with likely confined placental mosaicism. Figure 3 shows comparison of array CGH and NGS analyses for the 47,XXY case and the trisomy 18 case. An additional four cells with trisomy 21 are shown in Figure 4, all from a single case (NIPT511) in which all 11 fetal cells analyzed successfully showed trisomy 21. This case presented with cystic hygroma, and a blood sample was obtained from the mother at 13 weeks' gestation prior to a CVS procedure, which confirmed trisomy 21 by karyotype analysis. A case of a 2.7 Mb deletion of chromosome 15q25.2q25.3 (NIPT479) was also studied, from which two fetal cells were recovered and both showed the deletion (one cell shown in Figure 5). This demonstrates the potential for detecting relatively small deletions from individually isolated fetal cells.

**Figure 6:**
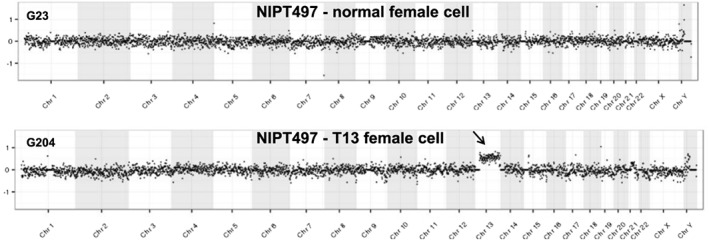
Demonstration of mosaicism with a normal and one trisomy 13 cell. NGS whole genome plots from two single trophoblastic cells derived from a female pregnancy suspected to have trisomy 13 by plasma‐based NIPT studies (subject 497). One of three normal cells is shown above, and the only trisomy 13 cell is shown below. Clinical cytogenetic studies on CVS tissue from this pregnancy showed a normal female FISH result and a 46,XX chromosome complement with no evidence of trisomy 13 mosaicism. NGS data is displayed as 1000 kb bins across the genome

Another notable case (NIPT497) was instructive regarding a likely occurrence of confined placental mosaicism. This case was recruited for study prior to a CVS procedure to confirm a positive plasma‐based NIPT result positive for trisomy 13. The CVS cytogenetic studies were normal, indicating a ‘false‐positive’ NIPT result. Of the nine fetal cells identified from this case, one cell demonstrated trisomy 13 and three additional cells had a normal copy number; the remaining five cells have not been analyzed (Figure 6). These data support the widely held suspicion that confined placental mosaicism is one source of positive plasma‐based NIPT results followed by normal amniocentesis or CVS.

## Discussion

These results presented here show that we can successfully isolate circulating trophoblasts and use them for noninvasive genome analysis by array CGH and NGS to demonstrate chromosomal aneuploidy and confirm fetal gender. There are important potential advantages of cell‐based compared to cell‐free NIPT if genome‐wide copy number analysis can be performed rather than using FISH as was often attempted in earlier years. First, any findings or abnormalities detected reflect the genome of the fetus and not that of the mother. Second, the ability to analyze multiple single cells allows for detection of mosaicism and for analysis of multiple independent cells. If this test were to be used clinically, it would be desirable to study three to five or more cells from a single sample. Third, although very deep sequencing of cell‐free DNA may eventually allow for detection of smaller CNVs, it is unlikely that this can ever be as cost effective and as reliable as analyzing pure fetal trophoblastic DNA. Finally, one long‐term goal should be to detect *de novo* point mutations, and this is predicted to be more reliable by testing multiple single fetal cells as compared to cell‐free DNA, where any *de novo* mutation may reflect a somatic change in the mother that is not present in the fetus. We attribute the success in recovering fetal cells reported to persistence in the face of many failures, meticulous attention to minor variations in the protocol, willingness to accept recovery of trophoblasts rather nucleated RBCs, and technological advances of the RareCyte system.

The current protocol includes considerable manipulation which could damage cells, and it is possible that more cells can be recovered with improved methods based on reports of one to six fetal cells per milliliter of maternal blood in some studies.^18^ Concern has been raised in the past about persistence of fetal cells from previous pregnancies. While there is clear evidence that male progenitor cells or lymphocytes can be recovered from the maternal blood as long as 27 years after a pregnancy,^30^ these cells are very rare and would not be expected to be positive for CK staining. Therefore, persistent cells from a previous pregnancy are extremely unlikely to be recovered by this method.

Another question to consider is whether analysis of trophoblast cells could result in positive findings because of confined placental mosaicism, which is detected by CVS in approximately 1–2% of viable pregnancies at 9–12 weeks of gestation.^31^, ^32^ We have already encountered one case reflecting confined placental mosaicism (NIPT497), and we anticipate that the frequency will be similar to that observed with current chromosomal analyses performed on CVS specimens and, therefore, would not be unfamiliar or of high frequency. Depending on whether the mechanism of mosaicism is similar to or different than that seen in direct and indirect CVS, sensitivity for detection of placental mosaicism may be lower or higher than that seen with CVS. The sensitivity for detection of mosaicism will depend on the number of cells studied, and in cases of aneuploidy results, it may be feasible to obtain a second blood sample and analyze 10–20 individual cells as evidenced by recovery of nine trisomy 21 cells from one blood sample. However, the frequency of confined placental mosaicism detected by this assay will need to be determined empirically through validation studies. One goal would be to reduce the risk of failing to detect mosaicism when it is present by analyzing multiple cells, perhaps three to five, individually by NGS or array. Any positive findings of even a single cell with aneuploidy potentially caused by mosaicism present in the placenta could be resolved definitively by follow‐up amniocentesis, as is currently recommended for mosaic CVS results. False negative placental mosaicism where CVS was normal but a child was born with aneuploidy is exceedingly rare in cultured cells but has been observed in direct analysis,^31^ and it would likely be rare with circulating trophoblasts as well, although this would likely depend on the number of cells analyzed in all routine cases.

An important question is whether cell‐based fetal copy number analysis can become a routine prenatal test offered to low‐risk as well as high‐risk pregnant women. It is known that current forms of noninvasive prenatal testing (NIPT) based on analysis of cell‐free DNA are not highly reliable for detecting subchromosomal abnormalities. One recent report using 3.5 million reads for cell‐free NIPT found 71.8% detection of variable sized subchromosomal copy number abnormalities and 55 false‐positives in 1476 samples.^33^ Another report using standard read depth for trisomy testing detected 15/18 samples with pathogenic rearrangements >6 Mb but only 2/10 samples with rearrangements <6 Mb.^34^ Based on our experience indicating that copy number variants (CNVs) can be detected at a resolution of 1 Mb in single lymphoblast cells,^24^ we are optimistic that a similar resolution can be obtained with single trophoblastic cells, although these cells are often subjected to fixation and/or permeabilization, and analysis of fetal trophoblastic cells from the mother's blood is more difficult than for unfixed cultured cells. Our current data demonstrate that a 2.7 Mb deletion could be detected from two individual single trophoblastic cells by NGS whereas a 1.2 Mb deletion was not detectable in any of four single cells by array CGH. While optimization of WGA methods could improve our copy number resolution, this 2.7 Mb deletion is well below the limits of resolution for currently available plasma‐based NIPT. The experience with various forms of pre‐implantation genetic screening on single cells or small pools of cells suggests the technical feasibility of such an approach.^35^, ^36^ There is evidence that single nuclei can be analyzed in batches of 48 or 96 using NGS with copy number detection at a resolution of 54 kb in 5–6 days,^37^, ^38^ but this resolution has not been shown with single cells subjected to fixation and permeabilization. Furthermore, it is not yet possible to definitively identify a cell as carrying the fetal genome without molecular confirmation. We therefore propose that any test should include robust genotyping of individual cells following WGA but prior to any pooling or further analysis, primarily so that maternal cells can be identified and eliminated. This is especially important with female pregnancies because the gender cannot be used as confirmation of fetal origin. We currently believe that single cell NGS for three to five cells may be the most attractive pathway for a robust and high throughput routine clinical test. We have made no estimates of false positive or false negative rates at this time. We are initiating a validation study in women undergoing amniocentesis or CVS with array CGH, and comparing that result to the results obtained from cell‐based NIPT using array CGH and NGS.

Perhaps, the most important factor in the feasibility of using cell‐based NIPT will be the false positive rate for CNVs that might lead to unnecessary invasive procedures. This will undoubtedly be linked to the level of resolution attempted in the microarray or NGS analysis with attempts to call smaller CNVs associated with a higher false positive rate. We are hopeful that cell‐based NIPT using array CGH, single nucleotide polymorphism (SNP) arrays, or copy number analysis by NGS can evolve to be as accurate as chromosomal microarrays performed after amniocentesis or CVS. As part of the cell analysis or cell selection, it may be necessary to take into account whether cells are in S phase or not, because S phase can cause unevenness of copy number for early and late replicating portions of the genome.^39^ Based on unpublished data for NGS of single cells in S phase (N. Navin, personal communication), we believe that it will be possible to identify cells in S phase by NGS and exclude them from clinical interpretation. We have not detected any fetal cells in S phase to date.

Based on the experience from preimplantation diagnosis, it should be possible to analyze single gene mutations with substantial advantages over cffDNA in the case of maternally inherited variants. It is reasonable to ask whether *de novo* point mutations could be detected using cell‐based NIPT. This approach is already available using cffDNA,^40^ but there is evidence that artifacts arising in amplification or sequencing can cause a high rate of false positives in this context.^41^ However, there is additional work suggesting that amplifying at least three individual cells or pools of cells and barcoding to focus on mutations in all three analyses can minimize the rate of false positive *de novo* mutations.^42^ Efforts to screen all pregnancies (including low risk) using exome‐ or genome‐wide sequencing for *de novo* point mutations is a difficult challenge, but this is likely to become feasible.^43^, ^44^, ^45^ *De novo* mutations in ~500 genes causing severe intellectual disability are believed to occur with a frequency of 3–5 per thousand,^46^ which would be 3–5 times higher than the incidence of Down syndrome.

Another question is whether cell‐based NIPT can become a high throughput test that can be offered to millions of women. We anticipate that NGS, as is currently used for high throughput cffDNA analysis, can ultimately be applied for sample analysis as an alternative to microarrays. We further believe that all of the enrichment, staining, picking, genotyping, amplification, and NGS steps can be automated. The current work‐flow is complex, the number of cells recovered is relatively low, and we have not performed a clinical validation study, but the core feasibility of cell‐based NIPT is demonstrated, and we believe that the limitations can be overcome. The cell isolation and WGA for the cases presented herein were performed at two different laboratories (Seattle and Houston, respectively), indicating some consistency of the methodology. We believe that this is the first demonstration of the successful use of array CGH and NGS whole genome sequencing to detect chromosomal abnormalities in fetal cells from maternal blood, including detection of a 2.7 Mb deletion.

## Supporting information

Supporting Figure S1: AccuCyte, CyteFinder, and CytePicker technology components. A. AccuCyte separation tube with float, sealing ring, and EpiCollector with inserted isolation tube. B. CyteSlide containing slide wells and CytePicker needle with ceramic tip. C. Open view of scanning compartment of CyteFinder digital fluorescence microscope emulating picking of fetal cells. Labeled are objectives (o), stage (s) holding CyteSlide (cs) and single‐cell collection tubes, and CytePicker needle (n)
Supporting Figure S2: Fetal trophoblastic cell enrichment and retrieval. A. The AccuCyte separation tube was used to process 6 mL of preserved blood. B. After centrifugation with the float (density 1.058–1.061 g/cm^3^) the blood separates, leaving the WBCs isolated between the tube wall and the float. A ring (B, arrowhead) is clamped to the outside of the tube, isolating the WBCs from the RBCs allowing the plasma to be aspirated off the top. C. A high‐density displacement fluid is added to the tube and centrifuged to displace the less dense WBCs above the float. D. A second ring (arrowhead) is clamped near the top of the float to keep WBCs above the float while they are being fixed, permeabilized, and stained (E). F. After staining, a heavy density fluid is mixed into the sample, and then a medium density fluid is layered through the EpiCollector® (arrowhead). An isolation tube (arrow) containing a light density fluid is then inserted into the EpiCollector®. The fully assembled device now contains a step‐gradient of density fluids. G. During centrifugation, the less dense cells (circles with arrows) are carried through the denser fluids leaving unbound antibody behind which acts as a ‘pseudo‐wash’ process. H. The isolation tube is removed from the devise, and the stained sample is loaded directly onto CyteSlides. I. The CyteSlides are loaded into the CyteFinder digital scanning microscope where fetal cells are identified for retrieval with the CytePicker module.

Supporting info item
