# Supplementary figures and images for: Evidence for feasibility of fetal trophoblastic cell‐based noninvasive prenatal testing
Source: Prenat Diagn. 2016 Oct 2;36(11):1009–19. doi: 10.1002/pd.4924 (PMC5129580; doi:10.1002/pd.4924)

## Slide 1
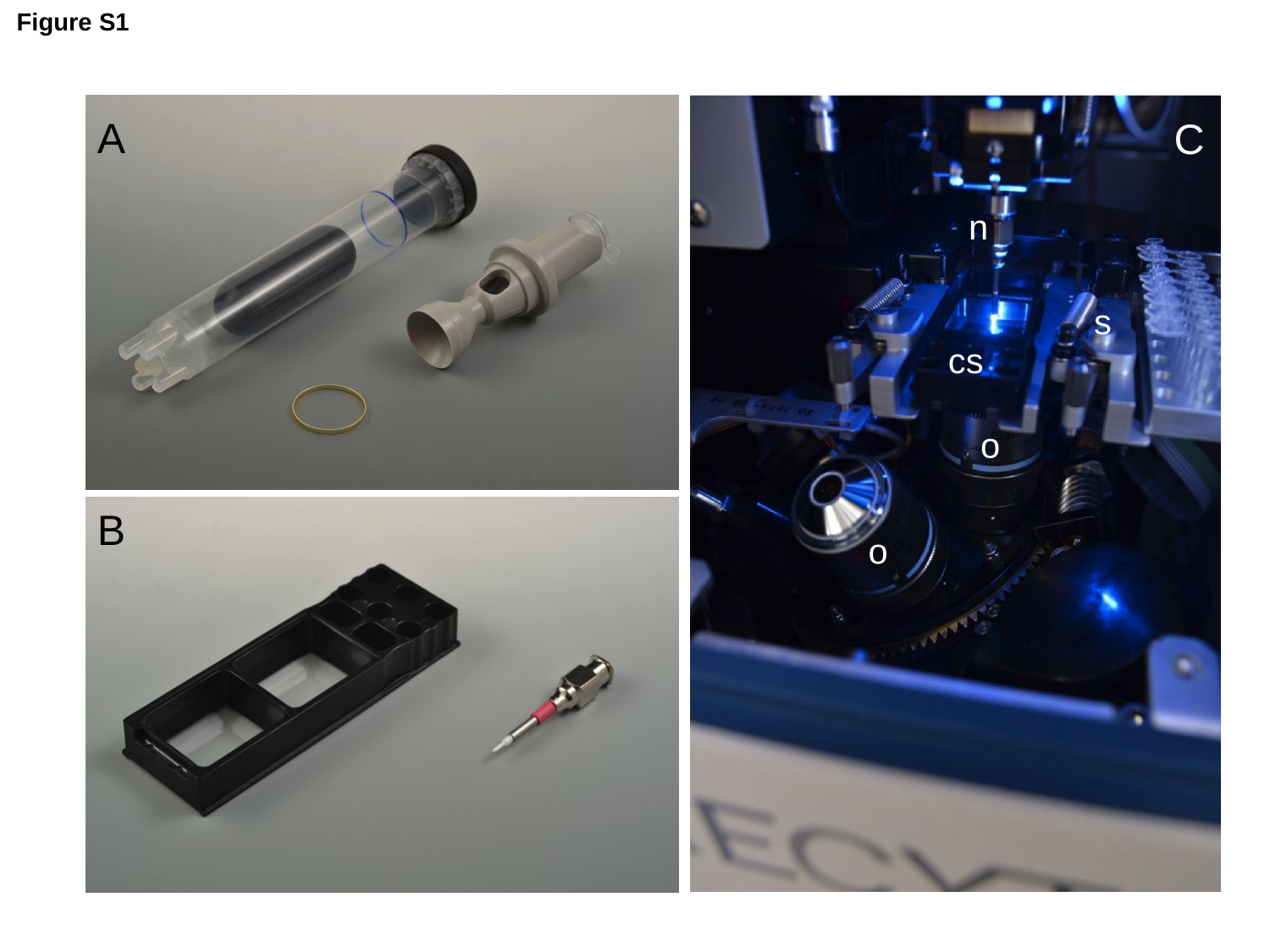

Figure S1
A
C
n
s
cs
o
B
o

## Slide 2
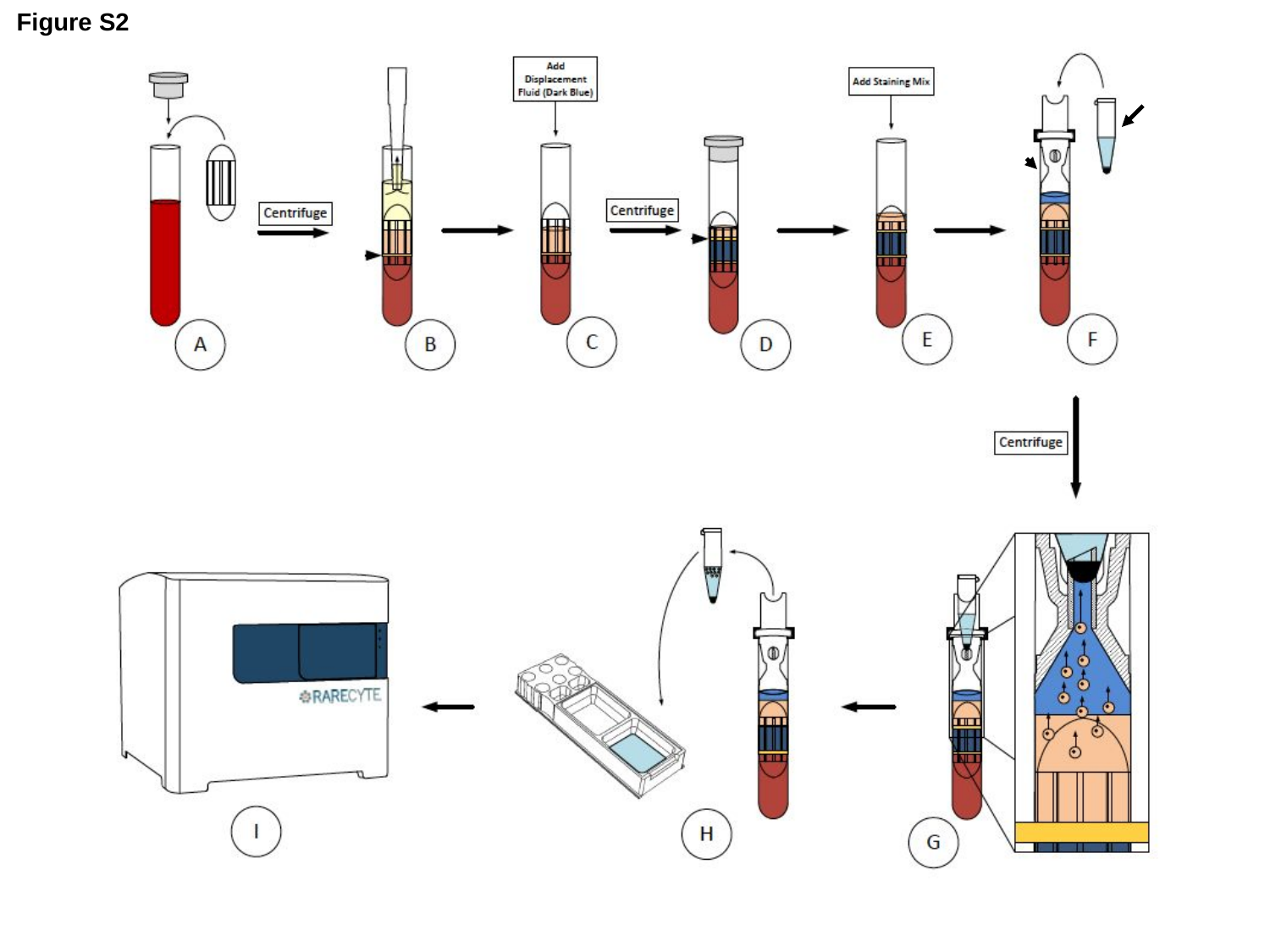

Figure S2

Supplement: Supplementary file 1 — Supporting info item [file PD-36-1009-s001.pptx]
